# Rapid and reliable alpha angle measurement using a smartphone‐based artificial intelligence system for cam‐type femoroacetabular impingement

**DOI:** 10.1002/jeo2.70418

**Published:** 2025-09-15

**Authors:** Masayoshi Saito, Hiroyuki Ogawa, Takuya Kusakabe, Naoyuki Hirasawa, Sachiyuki Tsukada

**Affiliations:** ^1^ Department of Orthopaedic Surgery Hokusuikai Kinen Hospital Ibaraki Japan

**Keywords:** alpha angle, artificial intelligence, cam‐type deformity, smartphone application

## Abstract

**Purpose:**

Manual alpha angle measurement for diagnosing cam‐type femoroacetabular impingement (FAI) is time‐consuming and variable. We hypothesised that a smartphone‐based artificial intelligence (AI) system would demonstrate a high correlation with the conventional manual method.

**Methods:**

We trained an AI model using 300 of the 45° Dunn view radiographs in a Python‐based environment. The model was implemented using a smartphone application that semi‐automatically extracted the femoral head from radiographs, detected at least four rim points, and calculated the femoral head centre and radius using a least‐squares method. It also identified the narrowest part of the femoral neck, and allowed manual adjustment of the alpha angle deviation. The images were directly captured and transferred without time loss. We evaluated 130 hips of patients with hip pain who underwent 45° Dunn view radiography. The alpha angle was measured using both the AI system and the manual method. Pearson correlation coefficient was used to assess agreement between the two methods. Measurement time and intra‐ and interobserver reliability were assessed using intraclass correlation coefficients (ICC).

**Results:**

Pearson correlation coefficient for alpha angle measurement between the two methods was 0.91, indicating a strong correlation. The average alpha angle measured by the AI system (52.7 ± 6.0°) was not significantly different from that of the conventional method (53.9 ± 6.5°, *p* = 0.12). The AI‐powered system showed intra‐ and interobserver agreement ICCs of 0.96 and 0.93, respectively, demonstrating excellent reliability. The mean measurement time was 5.0 ± 2.4 and 46.7 ± 10.1 s for the AI‐powered system and manual method, respectively, indicating significant time‐saving.

**Conclusion:**

The AI‐powered system showed a strong correlation with the manual method and significantly reduced measurement time. This smartphone‐based tool offers a rapid and reliable approach for real‐time assessment of alpha angles and may help standardise the evaluation of cam‐type FAI in clinical practice.

**Level of Evidence:**

Level III, retrospective comparative study.

**Abbreviations**
AI: artificial intelligence
CI: confidence interval
FAI: femoroacetabular impingement
ICC: intraclass correlation coefficient

## INTRODUCTION

Cam‐type femoroacetabular impingement (FAI) is a major cause of hip pain and restricted range of motion in young and active individuals. The alpha angle is a widely used radiographic parameter that quantifies the asphericity at the femoral head–neck junction, characterising cam morphology [1, 8, 12]. The alpha angle was originally defined on magnetic resonance imaging (MRI) using radial slices aligned to the femoral neck axis [14]. However, in routine clinical practice, the use of MRI or computed tomography (CT) is often limited by cost, time and availability. Consequently, plain radiographs remain the primary imaging modality for screening and diagnosing cam‐type FAI. Among these, the 45° Dunn view is widely adopted to visualise the anterosuperior region of the femoral head–neck junction, where cam deformities most commonly occur [17]. Although cam lesions are inherently three‐dimensional and dynamic, we focused on this view because it enables standardised measurement of the alpha angle at the most clinically relevant location. A previous study has reported that the alpha angle measured on the 45° Dunn view shows a high correlation (*r* = 0.81) with that obtained from radial MRI, and strong sensitivity (82%) for detecting cam lesions [17]. Despite its widespread use, alpha angle measurement on radiographs remains operator‐dependent and prone to interobserver variability, particularly when using manual techniques such as the circle of best‐fit method [15]. This variability can be especially pronounced in general clinical settings where experience levels differ. Modern technologies, including artificial intelligence (AI) and smartphone‐based applications, offer novel opportunities to improve both reproducibility and efficiency of radiographic measurements. However, no validated real‐time AI‐powered solution has yet been established for clinical use in measuring the alpha angle. This represents a critical gap in current orthopaedic diagnostic practice [15].

In this study, we developed and validated a smartphone‐based AI system for real‐time alpha angle measurement using 45° Dunn view radiographs. The primary objective was to assess the correlation between AI‐powered and conventional manual alpha angle measurements, and to evaluate the intra‐ and interobserver reliability, and measurement efficiency, of our AI‐powered system. We hypothesised that our AI‐powered system would demonstrate a high correlation with the conventional manual method.

## MATERIALS AND METHODS

### Study design, participants and outcomes

This retrospective cohort study was conducted at Hokusuikai Kinen Hospital following approval from the institutional review board (approval number: 2024‐095). We analysed 130 hips from consecutive patients who presented with hip pain and underwent 45° Dunn view radiography at our outpatient clinic between January 2024 and July 2024. The inclusion criteria were: (1) availability of adequate quality 45° Dunn view radiographs; (2) age between 18 and 60 years and (3) presence of clinical symptoms suggestive of cam‐type FAI. The exclusion criteria were: (1) radiographic evidence of hip osteoarthritis (Tönnis grade ≥2); (2) previous hip surgery; (3) history of trauma to the affected hip and (4) presence of inflammatory or rheumatologic joint diseases.

The primary outcome was the correlation of alpha angle measurements between the AI‐powered system and the conventional manual method. Secondary outcomes included intraobserver and interobserver reliability, and measurement time for both methods. As this was a diagnostic comparison study using static imaging data, no patient follow‐up was performed.

### Radiographic imaging

Radiology technologists obtained plain radiographic images using standardised techniques. A 45° Dunn view was obtained with the patient in the supine position with the symptomatic hip flexed at 45° and abducted at 20° in neutral rotation [4, 17].

### Development of the AI‐powered system

**Figure 1:**
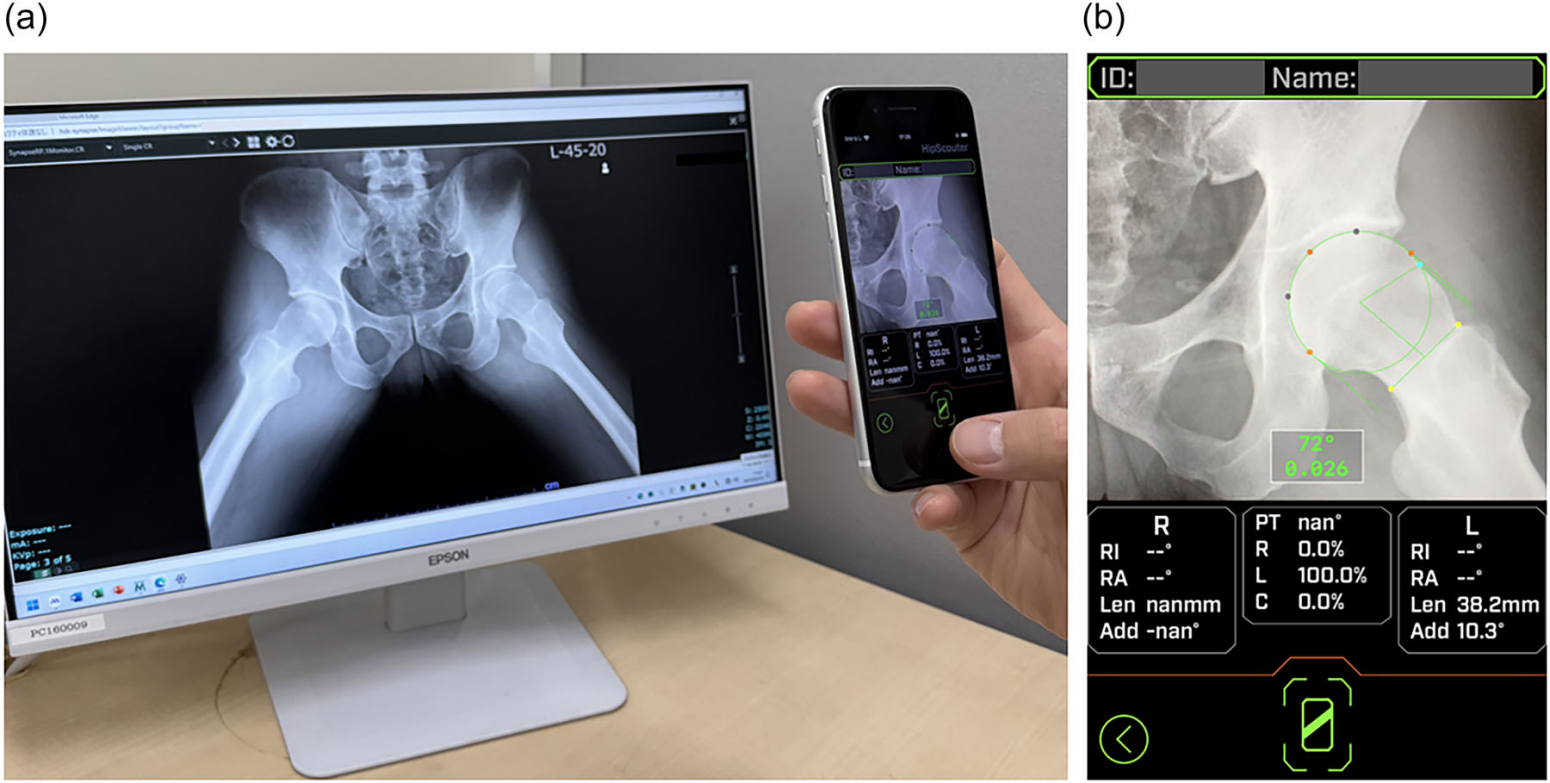
Artificial intelligence‐powered system for the immediate measurement of alpha angle. (a) Alpha angle is measured by capturing a 45° Dunn view radiograph using a smartphone camera. (b) Screenshot image of (a).

**Figure 2:**
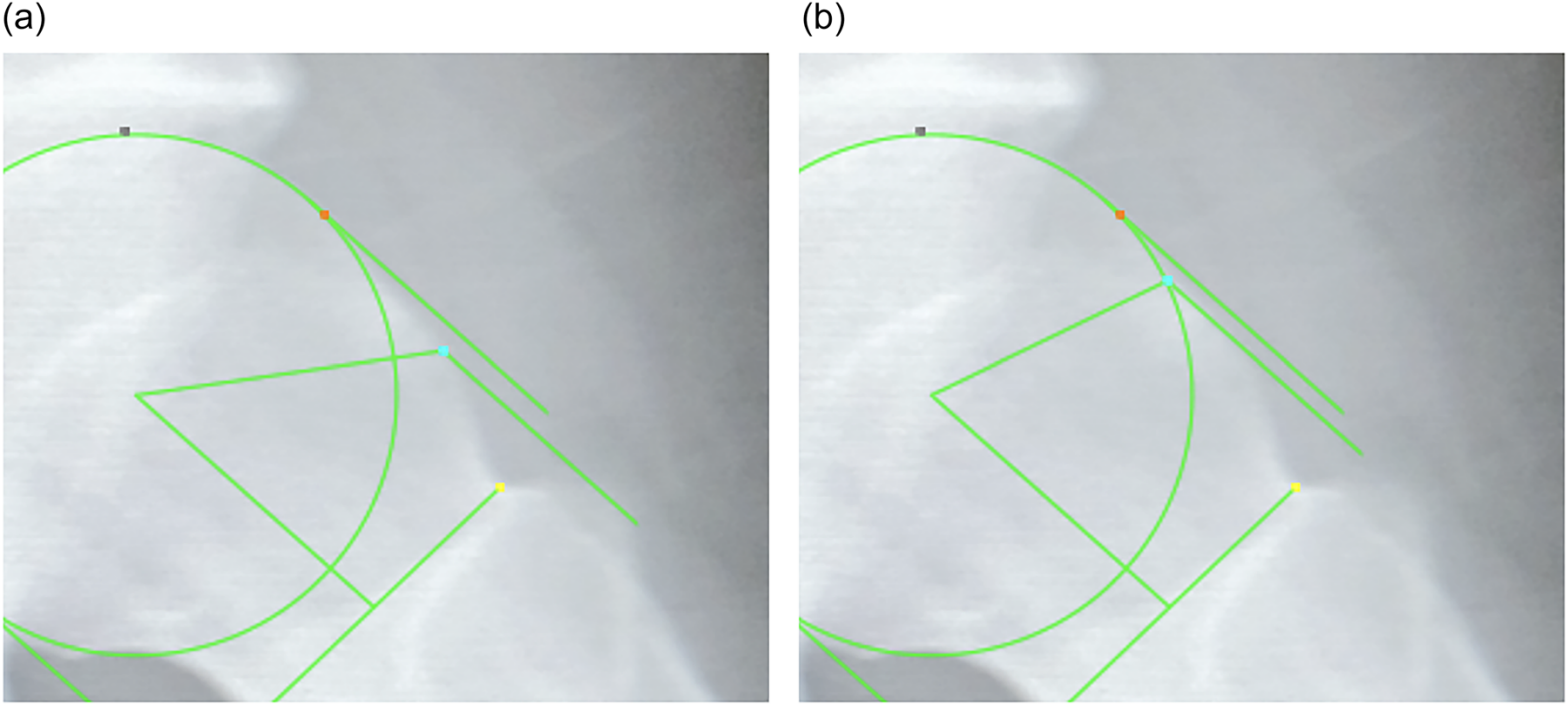
(a) The screen initially displayed by the artificial intelligence‐powered system (light blue point) reveals that the alpha angle deviation point is incorrect. (b) The alpha angle deviation point is then manually adjusted using the touch panel of the smartphone (light blue point).

We developed an AI‐powered system for semi‐automatic measurement of the alpha angle using a smartphone application (Cam Scouter, Figure 1). We collected 300 radiographs from patients who underwent 45° Dunn view imaging at our institution. These radiographs were consecutively selected, and images were excluded if they showed poor visualisation, improper positioning, or radiographic evidence of advanced osteoarthritis (Tönnis grade ≥2). The number of training images was determined based on previous experience in similar medical imaging studies and was considered sufficient for initial model development [18]. The AI model was trained using 300 of 45° Dunn view radiographs in a Python‐based environment. The AI model was developed using YOLOv5x, a high‐parameter version of the YOLOv5 architecture that has demonstrated improved performance in complex object detection tasks. This variant is particularly suitable for medical imaging applications that require precise localisation of anatomical features such as the femoral head and neck region. A total of 300 anonymized 45° Dunn view radiographs were used for training, and an independent set of 100 radiographs was used for validation. Model training was performed for 1000 epochs in a Python‐based environment using the PyTorch framework. All images were preprocessed and annotated using standardised bounding boxes encompassing the femoral head and neck regions. Training was performed using the default YOLOv5 training pipeline settings optimised for object detection tasks on medical imaging datasets. The system processes radiographic images by automatically detecting the femoral head region, extracting at least four rim points, and calculating the femoral head centre and radius using a least‐squares method. Additionally, it identifies the narrowest part of the femoral neck and allows the alpha angle deviation point to be manually adjusted using the touch panel of the smartphone (Figure 2). This semi‐automated process generally requires minimal manual adjustment. The alpha angle deviation point is the position on the femoral head where the head‐neck junction first departs from a best‐fitting circle around the femoral head. The smartphone application enables real‐time image capture and alpha angle measurement (Supporting Information S1).

### Measurement procedures

The evaluation procedure consisted of alpha angle measurement using both methods, assessment of agreement, observer reliability and measurement time. Alpha angle measurements were performed using both the AI‐powered system and the conventional manual method. A single observer, a board‐certified orthopaedic surgeon with 15 years of experience, evaluated the radiographs and measured the alpha angle using both methods. The manual method followed the approach of Notzli et al., in which the alpha angle was measured by identifying the centre of the femoral head and the point at which the femoral head contour deviated from a perfect circle [14]. We anonymized patient names in the hospital's Picture Archiving and Communication System, and exported postoperative 45° Dunn view pelvic radiographs as JPEG files onto a CD‐R. These images were then transferred to a computer equipped with software (Two‐Dimensional Template; Kyocera, Kyoto, Japan) for the conventional measurement of the alpha angles on 45° Dunn view radiographs. This manual measurement method has been established in a previous study using 45° Dunn view radiographs, which reported an intraclass correlation coefficient (ICC) between two independent observers of 0.93 (95% confidence interval [CI]: 0.87–0.96) [17]. In our AI‐powered system, the alpha angle was semi‐automatically calculated using the method described by Notzli et al., followed by image processing using the aforementioned smartphone application. The system was capable of two measurement modes: fully automated and semi‐automated. The fully automated process was defined as cases in which the alpha angle deviation point was correctly identified by the system without the need for manual adjustment. The semi‐automated process was defined as cases in which minor manual correction of the alpha angle deviation point was required using the smartphone's touch interface. Both measurement types were included in the measurement time analysis. The time required to measure the alpha angle was recorded for both methods. The measurement time was defined as the duration from the initiation of landmark identification to the completion of alpha angle determination. The duration was measured using a stopwatch by a third observer who was not involved in the measurements and was blinded to the study objectives. The mean measurement times were calculated and compared between the two methods.

### Statistical analysis

We conducted a power analysis using G*Power 3.1.9.7 (Heinrich Heine Universität, Düsseldorf, Germany), which indicated that at least 112 participants were required to detect a medium effect size (*r* = 0.3) with 90% power at a significance level of 0.05. Our final sample of 130 hips exceeded this threshold.

To assess validity, we calculated Pearson's correlation coefficients between the alpha angle measurements obtained by the AI‐powered system and those obtained manually. The strength of the correlation was categorised as negligible (<0.1), weak (0.1–0.39), moderate (0.4–0.69), strong (0.7–0.89), or very strong (>0.9). We also evaluated the absolute difference between the two methods.

For intraobserver reliability, a single observer performed three independent measurements with a minimum 4‐week interval to minimise recall bias. Interobserver reliability was assessed using measurements from two experienced orthopaedic surgeons working independently. ICCs were calculated for both intra‐ and interobserver reliability.

Measurement times were compared using an independent *t*‐test.

All analyses were performed using R software (version 4.2.2), and statistical significance was set at *p* < 0.05.

## RESULTS

### Correlation and reliability of alpha angle measurements

**Figure 3:**
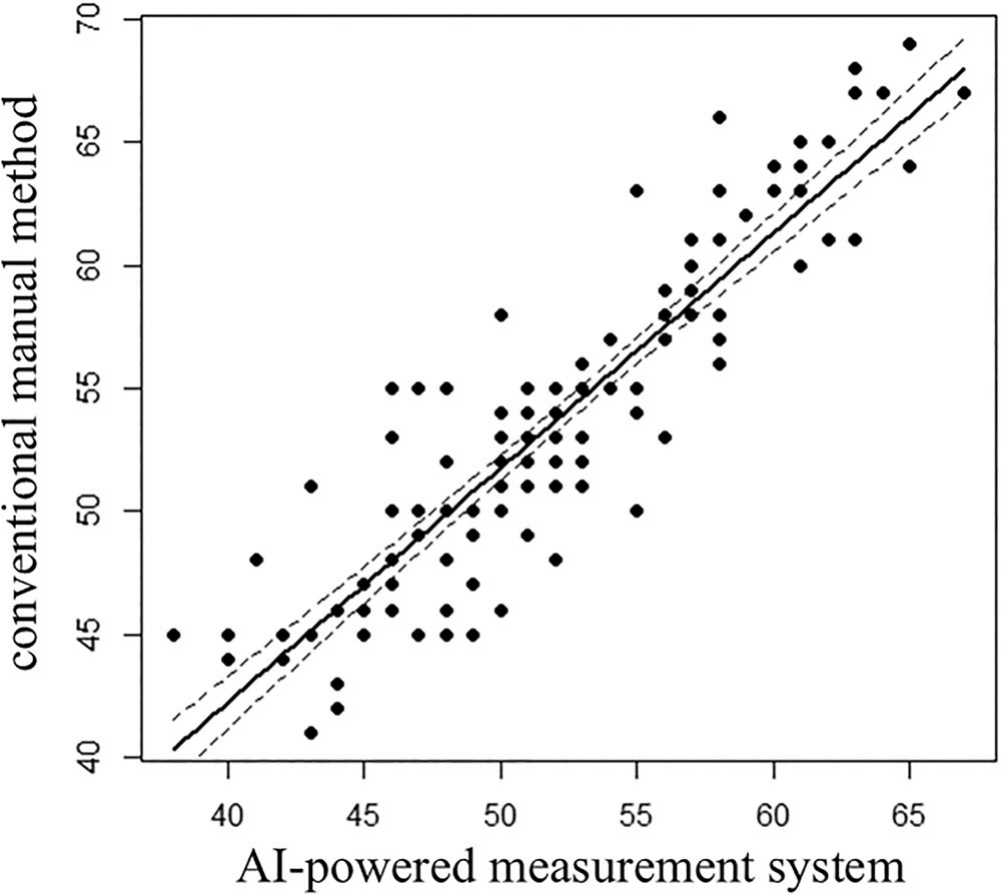
Correlation of alpha angles between the artificial intelligence‐powered measurement system and the conventional manual method. Pearson correlation coefficient = 0.91 (95% confidence interval: 0.87–0.93, *p* < 0.001).

Alpha angle measurements obtained using the AI‐powered system showed a very strong correlation with those from the manual method (*r* = 0.91, 95% CI: 0.87–0.93, *p* < 0.001, Figure 3). Tables 1 and 2 summarise the correlation and reliability results.

**Table 1:** Correlation and reliability of alpha angle measurements.

Outcome	Value	95% confidence interval
Pearson's correlation (AI vs. manual)	0.91	0.87–0.93
Mean absolute difference (AI vs. manual)	2.7 ± 2.0°	2.4–3.1°
Intraobserver ICC (AI)	0.96	0.94–0.97
Interobserver ICC (AI)	0.93	0.90–0.95
Mean absolute difference between observers	2.6 ± 2.0°	2.2–2.9°

*Note*: AI: AI‐powered system. Manual: conventional manual method.

Abbreviations: AI, artificial intelligence; ICC, intraclass correlation coefficient.

**Table 2:** Comparison of the alpha angle and measurement time between methods.

	Aartificial intelligence‐powered system	Conventional manual method	*p*‐Value
Alpha angle, degree	52.7 ± 6.0°	53.9 ± 6.5°	0.12
Measurement time, s	5.0 ± 2.4	46.7 ± 10.1	<0.0001

*Note*: Results are expressed as means ± standard deviation, unless otherwise indicated. *p*‐values were determined using Student's *t*‐test.

### Comparison of measurement time

The AI‐powered system measured the alpha angle in an average of 5.0 ± 2.4 s, significantly faster than the manual method (46.7 ± 10.1 s, *p* < 0.0001, Table 2). In 21 cases (16.2%), fully automatic measurement was completed in 1.0 s, while in the remaining 109 cases (83.8%), semi‐automatic measurement with minimal manual adjustment of the alpha angle deviation point took 3–10 s.

## DISCUSSION

In this study, we developed and validated an AI‐powered system for the semi‐automated measurement of the alpha angle using a smartphone. The system demonstrated a very strong correlation with conventional manual measurements, and excellent intra‐ and interobserver reliability. Additionally, it significantly reduced measurement time, with an average measurement duration of 5.0 s compared to 46.7 s with the conventional manual method. Manual alpha angle measurement is time‐consuming and prone to observer variability. Although conventional methods can achieve high interobserver reliability when performed by experienced orthopaedic surgeons, as demonstrated in our previous study (ICC = 0.93), such consistency may not be reproducible in routine clinical practice, particularly among less experienced clinicians. This study addresses this limitation by introducing an accessible smartphone‐based tool that provides reliable and efficient alpha angle measurements. The findings suggest that this AI‐powered system provides accurate, consistent and highly efficient alpha angle measurements, potentially improving the diagnostic efficiency of cam‐type FAI.

Our AI‐powered system reduced the measurement time by approximately 90%, which can significantly decrease the burden on clinicians. Manual measurement is time‐consuming because it requires precise identification of anatomical landmarks and careful angle determination [7, 15]. Although the system achieved full automation in a subset of cases, a manual correction step was necessary in approximately 84% of cases. However, the overall measurement time remained significantly shorter than that of the manual method, supporting the efficiency of the system, even in a semi‐automated workflow. A MRI‐based FAI study reported that it took approximately 11.2 min after training to manually measure the alpha angles in seven radial MRI slices, approximately 1.6 min (96 s) per image [6]. This exceeded that recorded in the present study, where the manual method took an average of 46.7 s. This time burden becomes particularly problematic in busy outpatient settings where efficiency and throughput are critical. The improvement in measurement time with our AI‐powered system could facilitate faster screening during outpatient consultations, particularly when multiple patients require alpha angle evaluation. Although both methods require less than a minute per case, the approximately 90% reduction in time becomes clinically relevant when scaled across many patients in high‐volume outpatient clinics. This time efficiency can help reduce fatigue, minimise bottlenecks and enable repeated assessments when needed. Given these advantages, this system has the potential to significantly enhance the workflow efficiency in orthopaedic clinics.

In addition to its speed, this system offers improved reproducibility. Manual measurement is subject to significant intra‐ and interobserver variability, with previously reported ICCs ranging from 0.46 to 0.98 and 0.45 to 0.95, respectively, depending on imaging modality and technique [2, 3, 6, 9, 16, 17]. This is because it is extremely subjective to correctly locate the centre of the femoral head (using a best‐fit circle) and to identify the exact points where the contour of the femoral head deviates from the perfect circle [6]. In our study, the AI‐powered system achieved ICCs of 0.96 and 0.93 for intra‐ and interobserver reliability, respectively and the variability of the measurements was equivalent to that of measurement methods reported in previous studies [3, 17]. Our AI‐powered system can accurately and rapidly extract four or more rim points and detect the precise centre of the femoral head. We believe that this contributes to its high intra‐ and interobserver reliability.

To the best of our knowledge, this study is among the first to apply AI‐powered image analysis to alpha angle measurement, which is a key diagnostic parameter for cam‐type impingement. Prior applications of AI in orthopaedic radiology have largely focused on postoperative assessments such as implant positioning in total hip arthroplasty [18], classification of femoral stem implants from radiographs [13], prediction of postoperative outcomes, complications and associated costs [11]. Our system differs in that it enables real‐time morphological assessment using a smartphone, making it accurate, reproducible and highly accessible.

### Clinical implications

This AI‐powered system enables rapid, reliable and reproducible alpha angle measurement using a smartphone, which could streamline the diagnostic workflow for cam‐type FAI, particularly in busy clinical settings where high diagnostic precision is required. Its speed and consistency make it a practical tool not only for orthopaedic hip surgeons, but also for general practitioners and sports physicians performing initial hip assessments. By addressing the long‐standing issue of interobserver variability in alpha angle measurement, this system may re‐establish the alpha angle as a reliable parameter across all stages of diagnosis, treatment planning, evaluation of treatment effects, follow‐up and academic research. Objective, standardised measurements independent of individual subjectivity enable accurate comparisons across institutions and readers in clinical studies, thus contributing to the standardisation and advancement of FAI research.

Furthermore, this study represents one of the first efforts to integrate real‐time AI‐powered measurement into routine clinical hip assessment. The ability to instantly obtain objective morphological data during outpatient evaluation may shift current workflows by reducing reliance on specialist radiological interpretation and improving diagnostic consistency across clinical settings.

Despite these promising results, certain limitations should be considered when interpreting the findings. The alpha angle reflects a two‐dimensional measurement and may not fully capture the three‐dimensional morphology of cam lesions, particularly outside the anterosuperior region [5]. Although we used the 45° Dunn view based on its strong clinical relevance and validated correlation with radial MRI, its ability to detect superior or posterior deformities remains limited [17]. Furthermore, all participants were recruited from a single outpatient clinic, which may restrict the generalisability of our results to more diverse or international patient populations. Further validation across multiple centres and imaging modalities would strengthen the applicability of our AI‐powered tool.

### Limitations

This study has some limitations. First, all radiographic images were obtained from a single institution, which may limit the generalisability of the findings. Second, although the AI‐powered system allows manual adjustments of the alpha angle deviation point, some cases may still require expert review to ensure optimal accuracy. Third, we did not compare AI measurements with 3D imaging modalities such as computed tomography or MRI, which could provide additional validation. Although we used the 45° Dunn view for alpha angle measurement due to its clinical relevance to the anterosuperior region, we acknowledge that cam deformity is a dynamic and three‐dimensional pathology [10]. Therefore, our measurements may not fully capture the complete extent of morphological variation. Fourth, the AI‐powered system does not currently provide automated warnings for incorrect imaging angles or the presence of other hip pathologies such as osteoarthritis. This may affect measurement accuracy in some cases and should be addressed in future system updates. Finally, although our system significantly reduced measurement time, its usability in diverse clinical settings and among different user groups (e.g., radiologists vs. orthopaedic surgeons) is unknown.

## CONCLUSION

The alpha angle measurements obtained using our AI‐powered system showed a very strong correlation with the conventional manual method and significantly reduced measurement time. While a single radiographic view cannot fully characterise the three‐dimensional nature of cam deformity, this system offers a practical and standardised tool for efficient assessment in outpatient settings. It may support early diagnosis and improve consistency in clinical evaluations.

## AUTHOR CONTRIBUTIONS

**Masayoshi Saito**: Writing—original draft; methodology; investigation; formal analysis; data curation; conceptualisation. **Hiroyuki Ogawa**: Writing—original draft; conceptualisation. **Takuya Kusakabe**: Investigation; formal analysis. **Naoyuki Hirasawa**: Writing—original draft; supervision. **Sachiyuki Tsukada**: Writing—original draft; conceptualisation.

## CONFLICT OF INTEREST STATEMENT

Sachiyuki Tsukada reports a relationship with Zimmer Biomet Japan that includes: consulting or advisory and speaking and lecture fees. The remaining authors declare no conflicts of interest.

## ETHICS STATEMENT

This study was approved by the institutional review board of Hokusuikai Kinen Hospital (Approval number: 2024‐095).

## Supporting information

The smartphone application automatically extracted the femoral head from radiographic images, detected at least four rim points, and calculated the femoral head center and radius using a least‐squares method. Additionally, it identifies the narrowest part of the femoral neck and allows the alpha angle deviation point to be manually adjusted using the touch panel of the smartphone. The smartphone application enables real‐time image capture and alpha angle measurement.

## Data Availability

The data that support the findings of this study are available on request from the corresponding author. The data are not publicly available due to privacy or ethical restrictions.
